# Supplementary material for: Small angle X-ray scattering and molecular dynamic simulations provide molecular insight for stability of recombinant human transferrin
Source: J Struct Biol X. 2019 Nov 30;4:100017. doi: 10.1016/j.yjsbx.2019.100017 (PMC7337065; doi:10.1016/j.yjsbx.2019.100017)
Supplement: Supplementary data 1 [file mmc1.docx]

## Appendix

Table A.1: SAXS - experimental details

| a) Sample details | | | | | |
| --- | --- | --- | --- | --- | --- |
|  | | | | Recombinant Transferrin^45^ | |
| Organism | | | | *Saccharomyces cerevisiae* | |
| Source | | | | Albumedix Ltd | |
| Extinction coefficient (A_280_, M^-1^cm^-1^) | | | | 85115 | |
| Molecular mass *M* from chemical composition (kDa) | | | | 75.135 | |
| b) SAXS data collection | | | | | |
| Instrument | P12 BioSAXS beamline (PETRAIII) | | | | BM29 BioSAXS |
| Date | 12.17 | 07.18 | 12.18 | | 12.17 |
| Detector | PILATUS2M | PILATUS6M | | | PILATUS1M |
| Wavelength (Å) |  | 0.124402 | | | 0.99 |
| Beam size (mm^2^) | 0.2 × 0.12 | | | | 0.7 x 0.7 |
| Detector distance (m) | 3.000 | | | | 2.867 |
| *q-*measurement range (nm^-1^) | 0.027-5.078 | 0.026 -7.288 | 0.0261-7.263 | | 0.039-4.9335 |
| Absolute scaling method | Comparison with scattering from pure H_2_O | | Comparison with scattering from BSA | | Comparison with scattering from pure H_2_O |
| Normalization | To transmitted intensity by beam-stop counter | | | | |
| Monitoring for radiation damage | Frame-by-frame comparison | | | | |
| Exposure time (s) | 20 x 0.05 | | 30 x 0.095 | | 10 x 1.00 |
| Sample configuration | Quartz glass capillary | | | | |
| Sample temperature (ºC) | 20 | | | | |
| c) Software employed for SAXS data reduction, analysis and interpretation | | | | | |
| SAS data reduction | *PRIMUSqt* ^22^ from *ATSAS* 2.8.3 ^46^ | | | | |
| Extinction coefficient estimate | *ExPaSy* ^47^ | | | | |
| Basic analyses: Guinier, *p*(*r*), *V*_P_ | *PRIMUSqt* ^22^ | | | | |
| Volume fractions of components | *OLIGOMER* ^22^ | | | | |
| Molecular graphics | *PyMOL* (version 1.8.2.3, Schrödinger, LLC) | | | | |

Table A.2: SAXS data collection – overview of the different physicochemical conditions tested.

| Buffer | pH | Additives | *c*_acdditives_ (mM) | *c*_rTrF_ (g/L) |
| --- | --- | --- | --- | --- |
| 10 mM histidine | 5.0 | - | - | 1.0, 2.0, 4.9, 6.8 and 9.4 |
|  |  | NaCl | 0, 35, 70 and 140 | 1.0 |
|  |  |  |  | 5.69, 5.25, 5.28 and 5.17 |
|  |  | Arginine-HCl | 0, 35, 70 and 140 | 3.5, 3.6, 3.7 and 3.7 |
|  |  | GuHCl | 0, 1000, 1500 and 2000 | 1.0, 1.0, 1.0 and 1.0 |
|  | 6.5 | - | - | 1.0, 2.0, 5.0, 7.0 and 10.0 |
|  |  | NaCl | 0, 35, 70 and 140 | 1.0, 1.0, 1.0 and 0.9 |
|  |  |  |  | 5.53, 5.20, 4.98 and 5.02 |
| 10 mM acetate | 4.0 | - | - | 0.69, 1.87, 4.80, 6.73 and 9.65 |
|  | 5.0 | - | - | 1.8, 3.5, 8.5, 12.0 and 17.5 |
|  |  | Arginine-HCl | 0, 35, 70 and 140 | 1.5, 1.3, 1.2 and 1.1 |
| 10 mM tris | 8.0 | - | - | 1.0, 2.0, 4.7, 6.4 and 9.0 |
|  |  | NaCl | 0, 35, 70 and 140 | 1.0, 1.0, 0.9 and 1.0 |

Table A.3: Structural parameters derived from SAXS experiments

|  | *c*_rTrF_ (g/L) | *c*_NaCl_ (mM) | *c*_arginine_  (mM) | Guinier | | *p*(*r*) | | | Apparent *MW* (kDa) | |
| --- | --- | --- | --- | --- | --- | --- | --- | --- | --- | --- |
|  |  |  |  | *I*(0)/*c* | *R*_g_ (nm) | *I*(0)/*c* | *R*_g_ (nm) | *D*_max_ (nm) | Guinier | *p*(*r*) |
| 10 mM acetate pH 4.0 | 1.00 | - | - | - | - | 14310* | 3.67 | 13.60 | - | 96 |
|  | 2.10 | - | - | - | - | 13620* | 3.46 | 12.00 | - | 91 |
|  | 4.80 | - | - | - | - | 13500* | 3.40 | 12.00 | - | 90 |
|  | 6.69 | - | - | - | - | 13090* | 3.30 | 11.57 | - | 87 |
|  | 9.72 | - | - | - | - | 12500* | 3.19 | 10.89 | - | 83 |
| 10 mM acetate pH 5.0 | 1.76 | - | - | 0.060 | 3.52 | 0.060 | 3.54 | 11.36 | 83 | 83 |
|  | 3.53 | - | - | 0.075 | 3.55 | 0.060 | 3.57 | 11.90 | 90 | 83 |
|  | 8.46 | - | - | 0.062 | 3.37 | 0.060 | 3.42 | 11.00 | 86 | 83 |
|  | 11.96 | - | - | 0.061 | 3.34 | 0.060 | 3.39 | 11.00 | 84 | 83 |
|  | 17.48 | - | - | 0.060 | 3.27 | 0.060 | 3.33 | 10.70 | 83 | 83 |
|  | 1.49 | - | 0 | 0.064 | 3.45 | 0.060 | 3.53 | 12.10 | 89 | 83 |
|  | 1.32 | - | 35 | 0.070 | 3.86 | 0.080 | 3.94 | 13.13 | 109 | 111 |
|  | 1.22 | - | 70 | 0.083 | 3.99 | 0.080 | 4.02 | 13.00 | 115 | 111 |
|  | 1.05 | - | 140 | 0.084 | 3.96 | 0.080 | 3.95 | 12.28 | 116 | 111 |
| 10 mM histidine pH 5.0 | 1.00 | - | - | 0.063 | 3.36 | 0.060 | 3.29 | 9.68 | 87 | 83 |
|  | 1.98 | - | - | 0.064 | 3.41 | 0.060 | 3.44 | 11.27 | 89 | 83 |
|  | 4.92 | - | - | 0.066 | 3.51 | 0.060 | 3.55 | 12.30 | 91 | 83 |
|  | 6.76 | - | - | 0.066 | 3.50 | 0.060 | 3.65 | 13.87 | 91 | 83 |
|  | 9.39 | - | - | 0.066 | 3.56 | 0.060 | 3.67 | 14.09 | 91 | 83 |
|  | 1.66 | - | 0 | 0.063 | 3.45 | 0.060 | 3.53 | 12.00 | 87 | 83 |
|  | 1.60 | - | 35 | 0.063 | 3.52 | 0.060 | 3.56 | 11.53 | 87 | 83 |
|  | 1.42 | - | 70 | 0.064 | 3.61 | 0.060 | 3.65 | 11.90 | 89 | 83 |
|  | 1.15 | - | 140 | 0.065 | 3.70 | 0.070 | 3.69 | 11.50 | 90 | 97 |
|  | 5.53 | 0 | - | 11976* | 3.53 | 12070* | 3.67 | 14.57 | 80 | 81 |
|  | 5.50 | 35 | - | 12919* | 3.72 | 12980* | 3.85 | 14.91 | 86 | 87 |
|  | 5.26 | 70 | - | 13824* | 3.95 | 13880* | 4.09 | 16.70 | 92 | 93 |
|  | 5.07 | 140 | - | 14931* | 4.05 | 15160* | 4.31 | 17.00 | 100 | 101 |
| 10 mM histidine pH 6.5 | 1.00 | - | - | 62** | 3.37 | 63** | 3.40 | 11.00 | 69 | 70 |
|  | 2.00 | - | - | 64** | 3.35 | 65** | 3.41 | 11.20 | 71 | 72 |
|  | 5.00 | - | - | 66** | 3.20 | 67** | 3.29 | 11.52 | 73 | 74 |
|  | 7.00 | - | - | 66** | 3.19 | 67** | 3.29 | 11.59 | 73 | 74 |
|  | 10.00 | - | - | 64** | 3.14 | 66** | 3.27 | 11.56 | 71 | 73 |
|  | 5.26 | 0 | - | 11279* | 3.29 | 11410* | 3.41 | 12.60 | 75 | 76 |
|  | 5.20 | 35 | - | 11592* | 3.47 | 11670* | 3.61 | 14.40 | 77 | 78 |
|  | 5.20 | 70 | - | 11288* | 3.37 | 11590* | 3.56 | 14.00 | 75 | 77 |
|  | 4.92 | 140 | - | 11703* | 3.58 | 11719* | 3.65 | 15.00 | 78 | 78 |
| 10 mM tris pH 8.0 | 1.00 | - | - | 0.059 | 3.54 | 0.060 | 3.47 | 11.00 | 82 | 83 |
|  | 1.95 | - | - | 0.057 | 3.40 | 0.060 | 3.41 | 11.00 | 79 | 83 |
|  | 4.71 | - | - | 0.055 | 3.22 | 0.050 | 3.30 | 11.65 | 76 | 69 |
|  | 6.37 | - | - | 0.054 | 3.18 | 0.050 | 3.26 | 11.00 | 75 | 69 |
|  | 8.98 | - | - | 0.053 | 3.13 | 0.050 | 3.24 | 10.93 | 73 | 69 |

Note: *DESY – BSA calibration; ** - data collected in ESRF

Table A.4: SEC-MALS experiments - overview of molecular weight (*MW*) and %Area at different pH and salt concentrations

| 10 mM His pH 5.0 | | 10 mM His pH 6.5 | | 10 mM Tris pH 8.0 | | Oligomeric state |
| --- | --- | --- | --- | --- | --- | --- |
| *M*W (kDa) | %Area | *MW* (kDa) | %Area | *MW* (kDa) | %Area |  |
| 0 mM NaCl | | | | |  |  |
| 79.1 | 85.2 | 75.3 | 85.4 | 74.9 | 85.2 | **Monomer** |
| 157.3 | 12.6 | 152.2 | 12.3 | 149.3 | 12.5 | **Dimer** |
| 241.6 | 1.9 | 240.3 | 1.9 | 229.0 | 1.9 | **Trimer** |
|  | | **70 mM NaCl** | |  | |  |
| 81.0 | 83.9 | 75.0 | 85.3 | 74.8 | 85.4 | **Monomer** |
| 157.5 | 14.0 | 148.9 | 12.5 | 148.7 | 12.4 | **Dimer** |
| 241.5 | 1.9 | 226.3 | 1.9 | 227.7 | 1.9 | **Trimer** |
|  | | **140 mM NaCl** | |  | |  |
| 83.8 | 86.0 | 75.0 | 85.5 | 74.9 | 85.7 | **Monomer** |
| 167.6 | 11.8 | 149.2 | 12.2 | 149.7 | 12.2 | **Dimer** |
| 267.3 | 1.7 | 225.5 | 1.9 | 230.6 | 1.8 | **Trimer** |

Table A.5: *OLIGOMER* data analysis

|  | *c*_rTrF_ (g/L) | *c*_NaCl_ (mM) | *c*_arginine_  (mM) | Open | Partially open | Closed | Dimer | ꭓ2 |
| --- | --- | --- | --- | --- | --- | --- | --- | --- |
| 10 mM acetate pH 4.0 | 1.00 | - | - | 0.04 (±0.04) | 0.60 (±0.05) | 0.15 (±0.02) | 0.201 (±0.006) | 1.05 |
|  | 2.10 | - | - | 0.08 (±0.02) | 0.62 (±0.03) | 0.12 (±0.01) | 0.178 (±0.004) | 1.04 |
|  | 4.80 | - | - | 0.122 (±0.009) | 0.59 (±0.01) | 0.116 (±0.006) | 0.176 (±0.002) | 1.21 |
|  | 6.69 | - | - | 0.156 (±0.007) | 0.550 (±0.009) | 0.139 (±0.005) | 0.156 (±0.002) | 1.27 |
|  | 9.72 | - | - | 0.19 (±0.01) | 0.52 (±0.02) | 0.178 (±0.009) | 0.109 (±0.003) | 1.09 |
| 10 mM acetate pH 5.0 | 1.76 | - | - | - | 0.38  (± 0.03) | 0.41  (± 0.04) | 0.236  (± 0.005) | 0.98 |
|  | 3.53 | - | - | - | 0.34  (± 0.02) | 0.43  (± 0.01) | 0.230  (± 0.004) | 1.01 |
|  | 8.56 | - | - | 0.01  (± 0.02) | 0.30  (± 0.03) | 0.50  (± 0.02) | 0.186  (± 0.004) | 1.03 |
|  | 11.96 | - | - | 0.007  (± 0.017) | 0.31  (± 0.02) | 0.51  (± 0.01) | 0.173  (± 0.003) | 0.96 |
|  | 17.48 | - | - | 0.06  (± 0.01) | 0.23  (± 0.02) | 0.558  (± 0.010) | 0.150  (± 0.002) | 1.03 |
|  | 1.49 | - | 0 | - | 0.38 (±0.05) | 0.41 (±0.06) | 0.207 (±0.006) | 0.99 |
|  | 1.32 | - | 35 | - | 0.57 (±0.03) | - | 0.434 (±0.003) | 1.27 |
|  | 1.22 | - | 70 | 0.04 (±0.004) | 0.48 (±0.04) | - | 0.480 (±0.004) | 1.42 |
|  | 1.05 | - | 140 | - | 0.489 (±0.005) | - | 0.511 (±0.004) | 1.62 |
| 10 mM histidine pH 5.0 | 1.00 | - | - | - | 0.52 (±0.02) | 0.28 (±0.02) | 0.206 (±0.002) | 1.15 |
|  | 1.98 | - | - | - | 0.68 (±0.01) | 0.121 (±0.014) | 0.199 (±0.002) | 1.21 |
|  | 4.92 | - | - | - | 0.551 (±0.006) | 0.234 (±0.006) | 0.215 (±0.001) | 1.52 |
|  | 6.76 | - | - | - | 0.519 (±0.005) | 0.258 (±0.005) | 0.223 (±0.001) | 1.68 |
|  | 9.39 | - | - | - | 0.509 (±0.006) | 0.261 (±0.004) | 0.229 (±0.001) | 2.41 |
|  | 5.53 | 0 | - | - | 0.578 (±0.004) | 0.201 (±0.004) | 0.221 (±0.001) | 2.77 |
|  | 5.50 | 35 | - | - | 0.637 (±0.004) | 0.073 (±0.005) | 0.290 (±0.001) | 4.65 |
|  | 5.26 | 70 | - | - | 0.644 (±0.005) | 0.003 (±0.005) | 0.353 (±0.001) | 6.16 |
|  | 5.07 | 140 | - | - | 0.546 (±0.001) | - | 0.454 (±0.001) | 8.00 |
|  | 1.66 | - | 0 | - | 0.52 (±0.02) | 0.28 (±0.02) | 0.206 (±0.002) | 1.15 |
|  | 1.69 | - | 35 | - | 0.746 (±0.003) | - | 0.254 (±0.002) | 1.16 |
|  | 1.62 | - | 70 | - | 0.684 (±0.004) | - | 0.316 (±0.003) | 1.05 |
|  | 1.64 | - | 140 | - | 0.612 (±0.005) | - | 0.388 (±0.004) | 1.66 |
| 10 mM histidine pH 6.5 | 1.00 | - | - | - | - | 0.829 (±0.003) | 0.171 (±0.002) | 1.13 |
|  | 2.00 | - | - | - | - | 0.812 (±0.002) | 0.188 (±0.002) | 1.61 |
|  | 5.00 | - | - | - | - | 0.893 (±0.001) | 0.107 (±0.001) | 4.72 |
|  | 7.00 | - | - | - | - | 0.904 (±0.001) | 0.096 (±0.001) | 9.28 |
|  | 10.00 | - | - | - | - | 0.929 (±0.001) | 0.071 (±0.001) | 19.33 |
|  | 5.26 | 0 | - | - | - | 0.859 (±0.001) | 0.141 (±0.001) | 1.52 |
|  | 5.20 | 35 | - | - | - | 0.835 (±0.001) | 0.165 (±0.001) | 2.64 |
|  | 5.20 | 70 | - | - | - | 0.834 (±0.001) | 0.166 (±0.001) | 2.76 |
|  | 4.92 | 140 | - | - | - | 0.835 (±0.001) | 0.165 (±0.001) | 2.75 |
| 10 mM tris pH 8.0 | 1.00 | - | - | - | - | 0.807 (±0.004) | 0.193 (±0.003) | 1.07 |
|  | 1.95 | - | - | - | - | 0.831 (±0.002) | 0.169 (±0.002) | 1.07 |
|  | 4.71 | - | - | - | - | 0.875 (±0.001) | 0.125 (±0.001) | 1.24 |
|  | 6.37 | - | - | - | - | 0.956 (±0.001) | 0.044 (±0.001) | 9.23 |
|  | 8.98 | - | - | - | - | 0.945 (±0.001) | 0.055 (±0.001) | 4.02 |


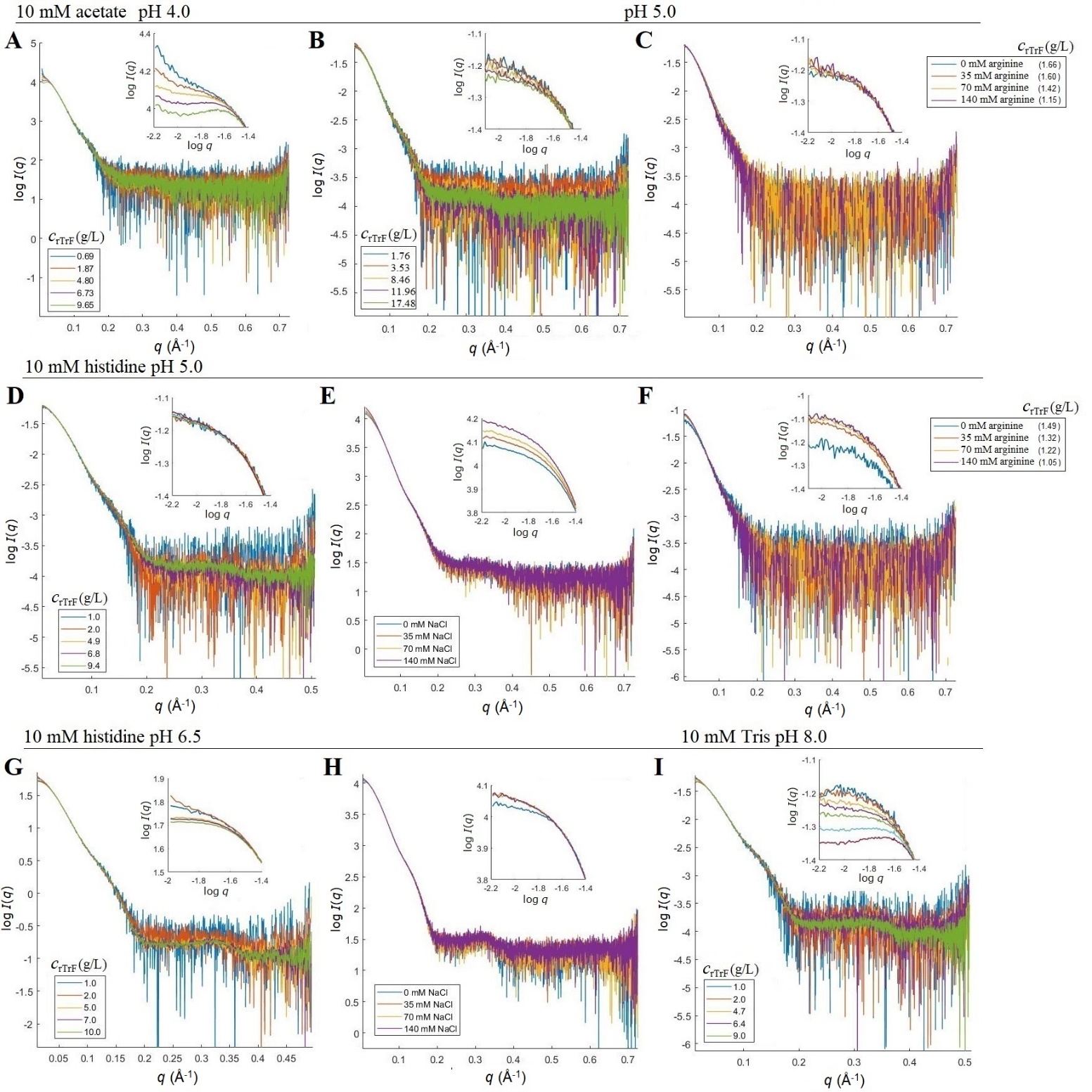


Figure A.1: SAXS scattering curves for concentration series at A – 10 mM acetate pH 4.0; B – 10 mM acetate pH 5.0; D – 10 mM histidine pH 5.0; G – 10 mM histidine pH 6.5; I – 10 mM tris pH 8.0. SAXS scattering curves with varying *c*_NaCl_ at E - 10 mM histidine pH 5.0 and H - 10 mM histidine pH 6.5 with *c*_rTrF_ around 5-5.5 g/L (see inset); and varying *c*_arginine_ at C (see inset) – 10 mM acetate pH 5.0 and F – 10 mM histidine pH 5.0 with *c*_rTrF_ around 1-1.5 g/L (see inset).


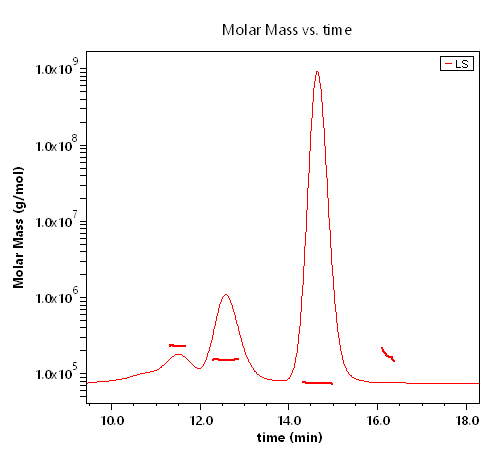


Figure A.2: SEC-MALS result at 10 mM histidine pH 5.0


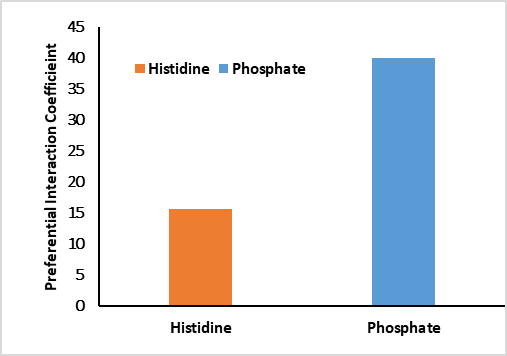


Figure A.3: Preferential interaction coefficient (PIC) of histidine and phosphate to rTrF at pH 6.5. The tendency of phosphate to bind to rTrF is significantly higher than for histidine; PIC_phosphate_~40 *vs* PIC_histidine_~15.


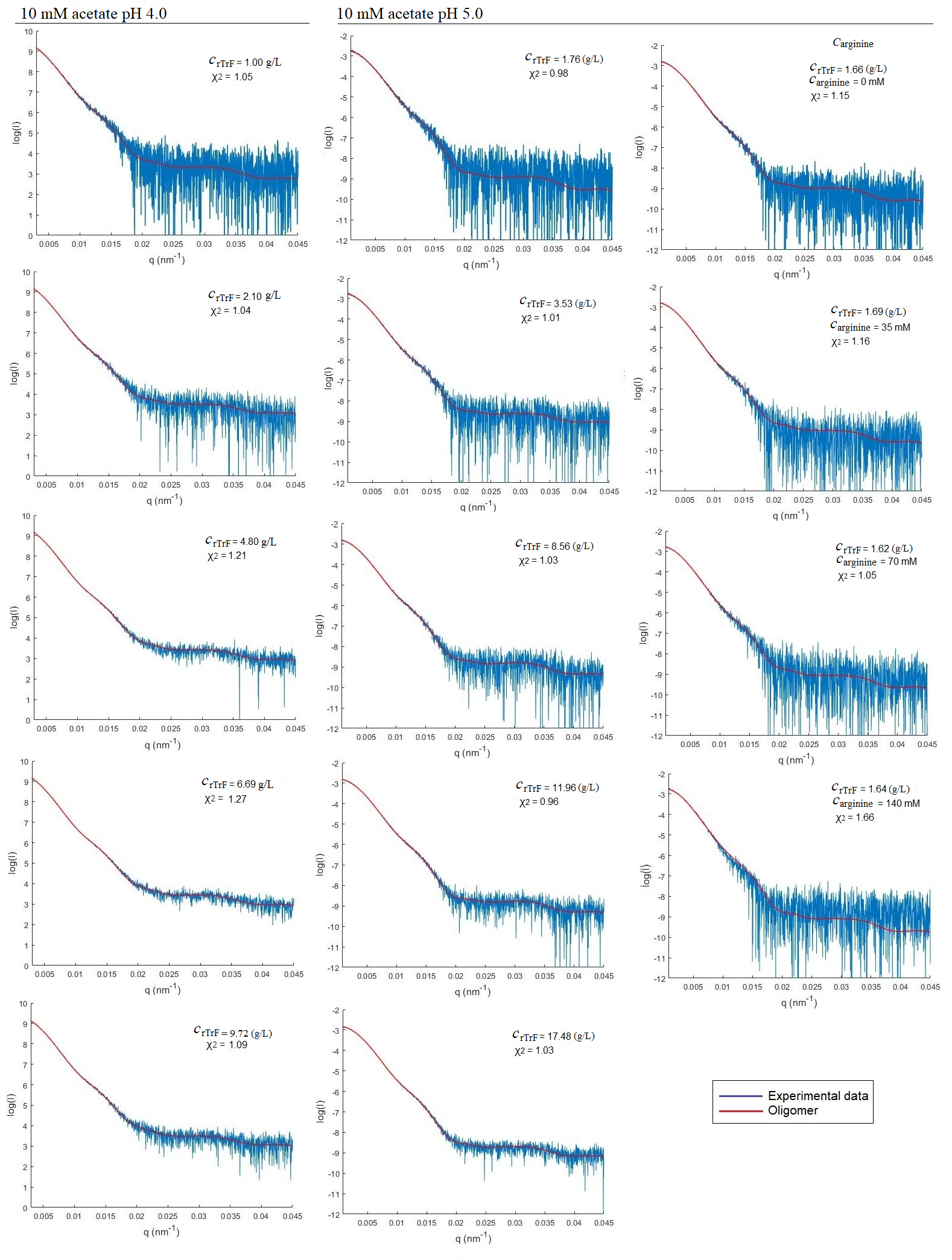


**A B C**

**D E F**

**G H I**

**J K L**

**M N**

Figure A.4: Fit plots from *OLIGOMER* analysis for *c*_rTrF_ at 10 mM acetate pH 4.0 (A, D, G, J, and M) and 10 mM acetate pH 5.0 (B, C, E, F, H, I, K, L, and N).


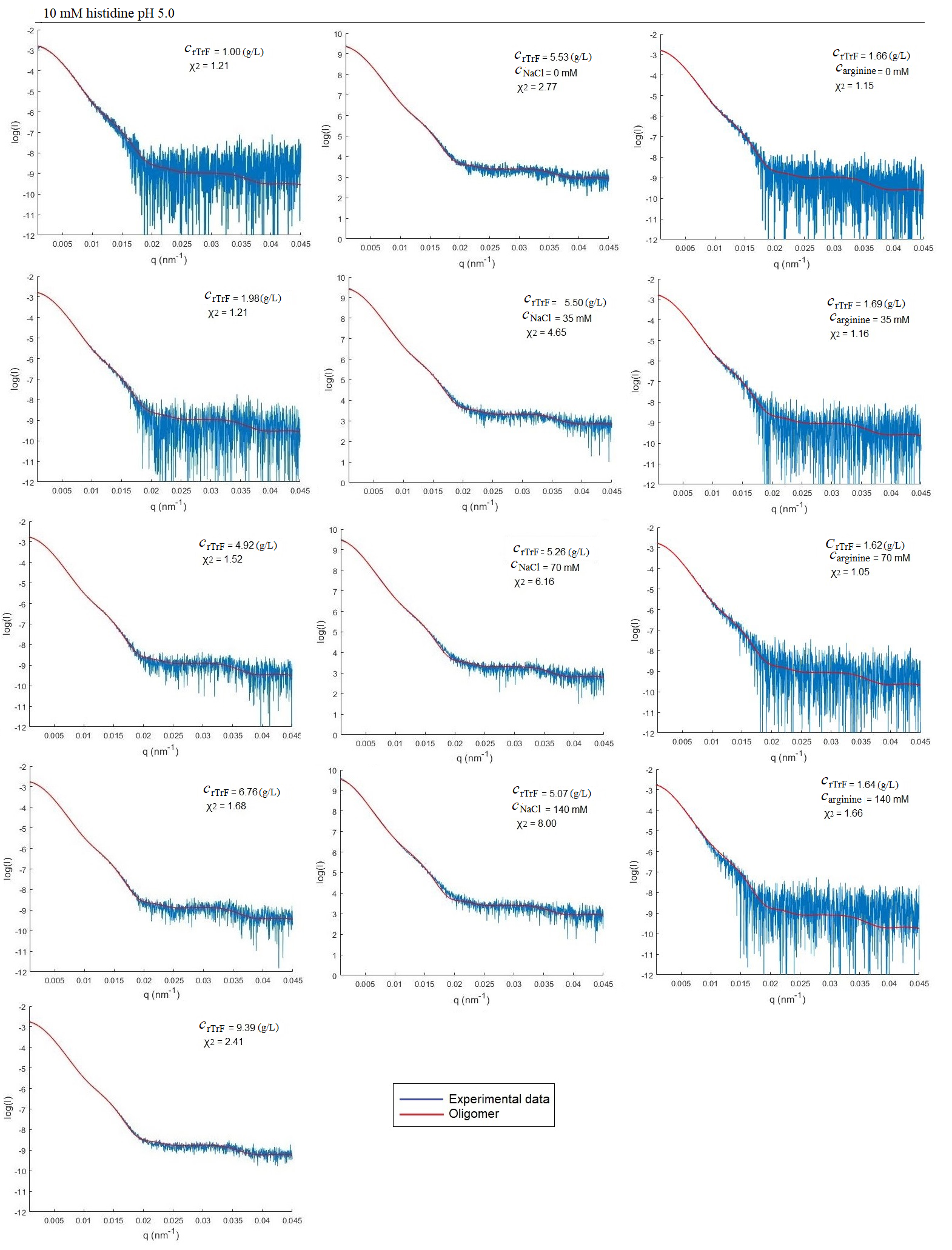


**A B C**

**D E F**

**G H I**

**J K L**

**M**

Figure A.5: Fit plots from *OLIGOMER* analysis for varying *c*_rTrF_ at 10 mM histidine pH 5.0 (A, D, G, J, and M), at 10 mM histidine pH 5.0 in the presence of NaCl (B, E, H, and K), and arginine (C, F, I, and L).


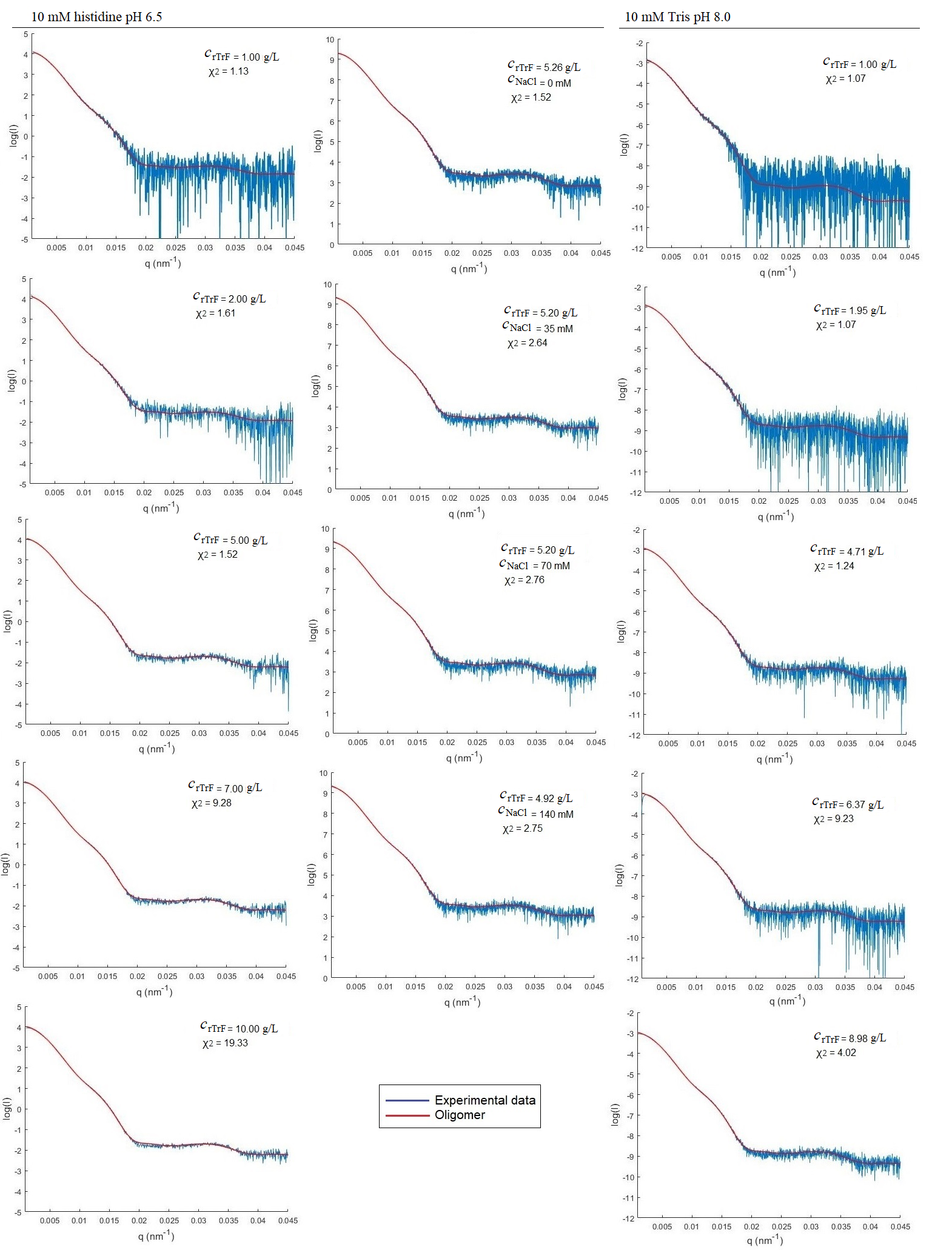


**A B C**

**D E F**

**G H I**

**J K L**

**M N**

Figure A.6: Fit plots from *OLIGOMER* analysis for varying *c*_rTrF_ at 10 mM histidine pH 6.5 (A, D, G, J, and M), at 10 mM histidine pH 5.0 in the presence of NaCl (B, E, H, and K), and at 10 mM tris pH 8.0 (C, F, I, L, and N).


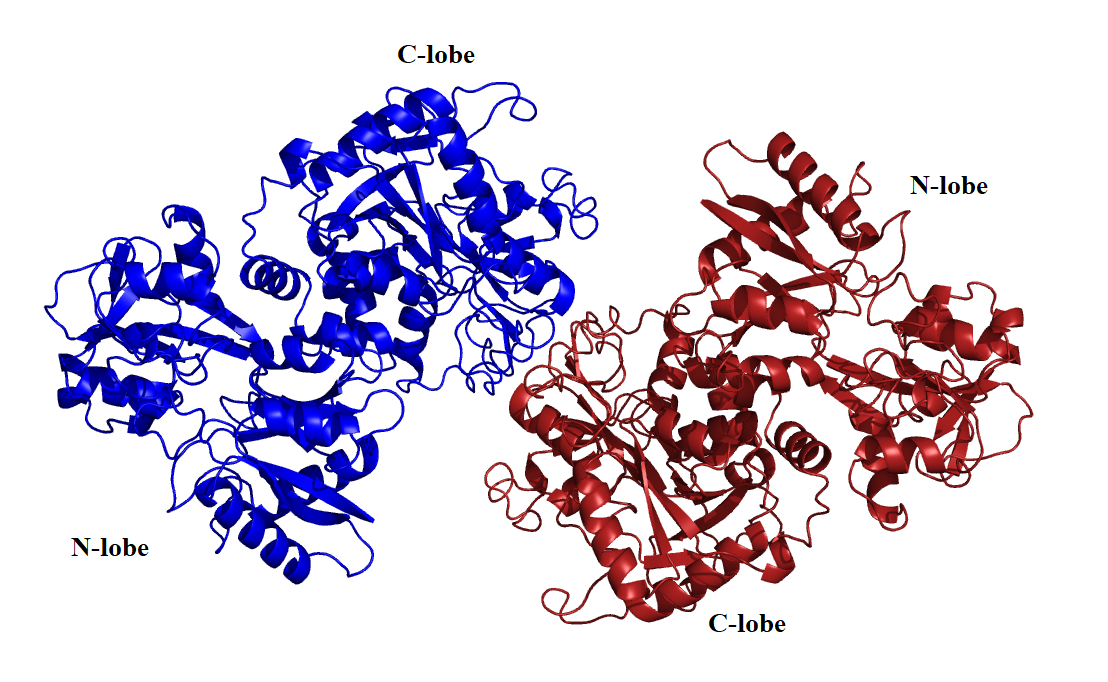


Figure A.7: Model of rTrF dimer obtained from *SASREFMX.*


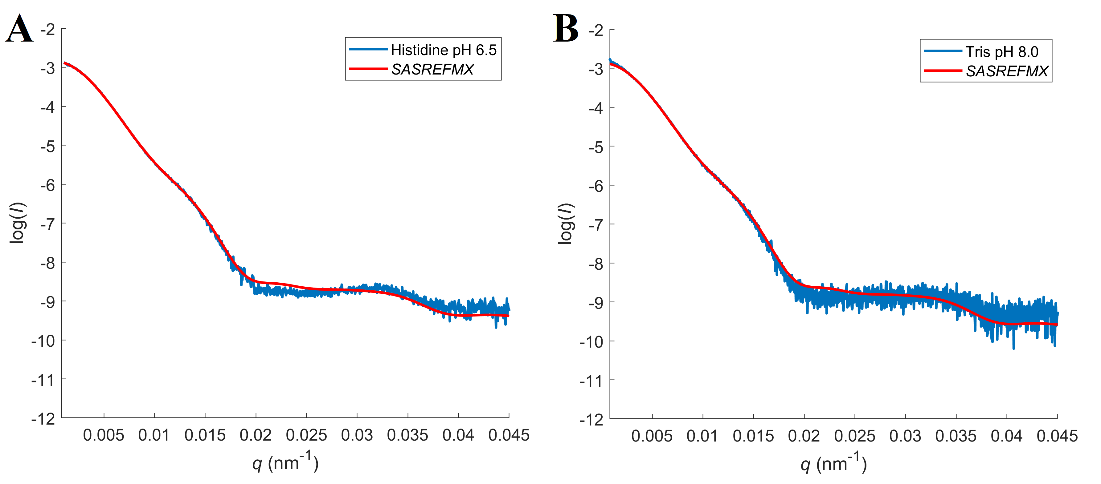


Figure A.8: Fit plots for *SASREFMX* (in red) on merged data (in blue) from A: histidine pH 6.5 and B: tris pH 8.0.

Table A.6: Static light scattering experimental details.

| ***c*_protein_ (g/mL)** | **Light scatter voltage (V)** | **Light scatter (V) - baseline (V)** | **Light Scatter Intensity (R_0_)** | **Kc/R** | **R/Kc** | **Second Viral Coefficient (B_22/_A_2)_** | **Molecular Weight (kDa)** |
| --- | --- | --- | --- | --- | --- | --- | --- |
| 0.003 | 0.5119 | 0.50188 | 4.97282E-05 | 1.3E-05 | 7.6E+04 | 0.00017 | 82.833 |
| 0.008 | 1.146 | 1.13598 | 0.000112557 | 1.5E-05 | 6.8E+04 |  |  |
| 0.011 | 1.487 | 1.47698 | 0.000146345 | 1.6E-05 | 6.4E+04 |  |  |
| 0.016 | 1.91 | 1.89998 | 0.000188257 | 1.7E-05 | 5.7E+04 |  |  |


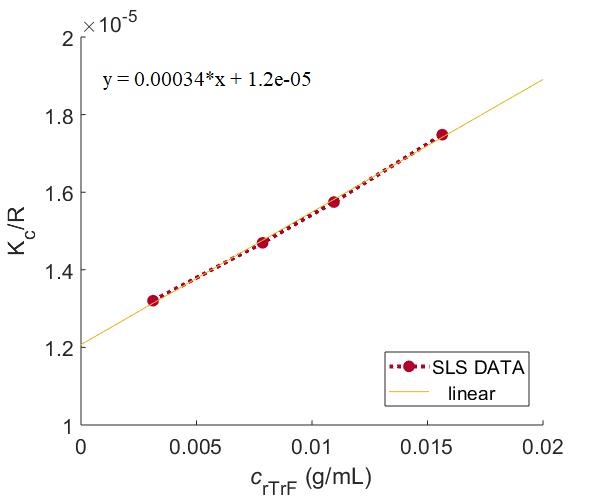


Figure A.9: Static light scattering plot (in red) with trend line (in yellow).


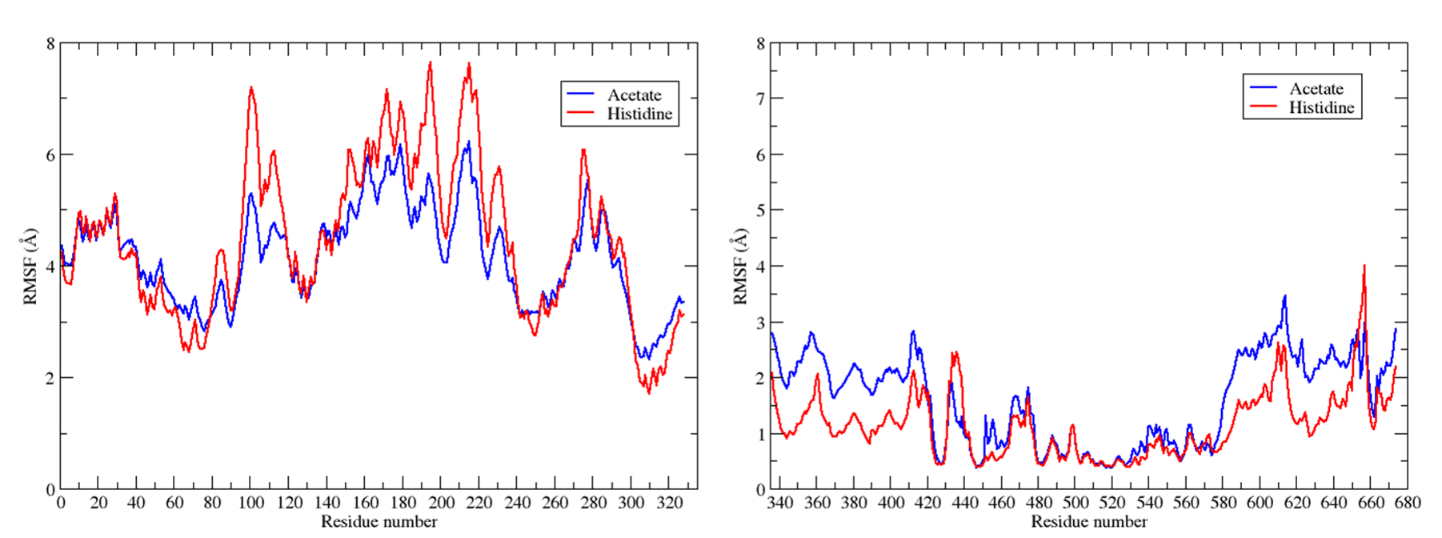


Figure A.10: RMSF plots for N-lobe (on the left) and C-lobe (on the right) in the presence of 140 mM acetate (in blue) and 140 mM histidine (in red).

Sequence^45^

VPDKTVRWCAVSEHEATKCQSFRDHMKSVIPSDGPSVACVKKASYLDCIRAIAANEADAVTLDAGLVYDAYLAPNNLKPVVAEFYGSKEDPQTFYYAVAVVKKDSGFQMNQLRGKKSCHTGLGRSAGWNIPIGLLYCDLPEPRKPLEKAVANFFSGSCAPCADGTDFPQLCQLCPGCGCSTLNQYFGYSGAFKCLKDGAGDVAFVKHSTIFENLANKADRDQYELLCLDNTRKPVDEYKDCHLAQVPSHTVVARSMGGKEDLIWELLNQAQEHFGKDKSKEFQLFSSPHGKDLLFKDSAHGFLKVPPRMDAKMYLGYEYVTAIRNLREGTCPEAPTDECKPVKWCALSHHERLKCDEWSVNSVGKIECVSAETTEDCIAKIMNGEADAMSLDGGFVYIAGKCGLVPVLAENYNKADNCEDTPEAGYFAVAVVKKSASDLTWDNLKGKKSCHTAVGRTAGWNIPMGLLYNKINHCRFDEFFSEGCAPGSKKDSSLCKLCMGSGLNLCEPNNKEGYYGYTGAFRCLVEKGDVAFVKHQTVPQNTGGKNPDPWAKNLNEKDYELLCLDGTRKPVEEYANCHLARAPNHAVVTRKDKEACVHKILRQQQHLFGSNVADCSGNFCLFRSETKDLLFRDDTVCLAKLHDRNTYEKYLGEEYVKAVGNLRKCSTSSLLEACTFRRP
